# Clinical features and outcomes of myelodysplastic syndrome patients with iron overload: a single-center retrospective study

**DOI:** 10.1186/s40001-025-02848-1

**Published:** 2025-07-09

**Authors:** Lei Huang, Yinxing Wang, Xinrui Zhang, Xintong Xu, Ziqi Zhang, XinRu Wu, Chunyan Liu, Rong Fu

**Affiliations:** 1 Department of Hematology, Tianjin Medical University General Hospital, 154 Anshan Street, Heping District, Tianjin, 300052 China; 2 Tianjin Key Laboratory of Bone Marrow Failure and Malignant Hemopoietic Clone Control, Tianjin, 300052 China; 3 Tianjin Institute of Hematology, Tianjin, 300052 China

**Keywords:** Myelodysplastic syndrome, Iron overload, Hematopoiesis, Immune index, Prognosis

## Abstract

**Objectives:**

Most of the myelodysplastic syndromes (MDS) patients suffer from iron overload (IOL) due to ineffective hematopoiesis and repeated blood transfusions. IOL may affect the survival of MDS patients, but the related mechanism has not been fully clarified. We aimed to provide clinical evidence for the impact of IOL on the immunity and prognosis of MDS patients.

**Methods:**

The clinical features and outcomes of 144 patients with MDS between March 2019 and December 2023 were analyzed. Patients were classified into the IOL group (ferritin > 1000 ng/mL) and the non-iron overload (NIOL) group (ferritin ≤ 1000 ng/mL).

**Results:**

The median age of the patients was 64 (22–89) years, and IOL MDS patients had a poorer performance status. IOL MDS patients had significantly higher alanine aminotransferase and blood glucose levels. White blood cell counts and hemoglobin levels were significantly lower in IOL MDS patients. Meanwhile, the proportion of erythrocyte and megakaryocyte counts were significantly decreased in MDS patients with IOL. The incidence of 7q- chromosome abnormalities in IOL group was significantly higher. The level of Interleukin-6 was markedly elevated in patients with IOL MDS, accompanied by significant abnormalities in dendritic cells. Survival analysis indicated that IOL MDS patients had a shorter survival duration. Age ≥ 60 years, ferritin > 1000 ng/mL, complex chromosomal abnormalities, and gene mutations in *TP53* and *RUNX1* were independent adverse prognostic factors for MDS patients.

**Conclusions:**

IOL MDS patients exhibit poorer performance status and organ function, with hematopoietic and immune function abnormalities potentially affecting their survival.

**Supplementary Information:**

The online version contains supplementary material available at 10.1186/s40001-025-02848-1.

## Introduction

Approximately 80–90% of myelodysplastic syndrome (MDS) patients have anemia at the beginning of the disease, they often suffer from iron overload (IOL) due to ineffective erythropoiesis and repeated blood transfusions [1]. The most common causes of non-leukemia-related death in IOL MDS patients are cardiac complications, liver damage and endocrine dysfunction [2–4]. IOL can induce manifestations of MDS and ineffective erythropoiesis in mice with Nrf2 deficiencies [5]. However, iron chelation therapy (ICT) improved ineffective erythropoiesis in these mice [6]. MDS patients are typically older, and limited treatments are available to prolong their life. Approximately half of the MDS patients eventually develop IOL. A recent meta-analysis suggested that ICT treatment was associated with a survival advantage in MDS patients, reducing the risk of transformation to acute myeloid leukemia (AML), and improving mortality rates in lower risk IOL MDS patients [7]. The damage of organ and bone marrow hematopoiesis caused by IOL increases the mortality rate of patients with IOL MDS, and ICT is associated with the survival benefit of IOL MDS patients. However, the possible mechanisms affecting the survival of patients with IOL MDS has not been fully clarified.

Immune abnormalities, gene mutations and chromosomal abnormalities are involved in the pathogenesis of MDS. The impaired function of T lymphocytes leads to immune failure, loss of immune surveillance and clonal proliferation of tumor cells [8]. Dendritic cells (DC_S_) are antigen-presenting cells on T cells and thus activate them. Studies have shown that the antigens of apoptotic hematopoietic stem cells in lower risk MDS patients are presented by DCs, triggering the damage of T cells to normal hematopoietic stem cells and facilitating the malignant clonal expansion and the progression of MDS. The pro-inflammatory microenvironment in MDS promotes the failure of normal hematopoiesis and the proliferation of malignant clones, which is secondary to the excessive production of inflammatory cytokines, affecting bone marrow hematopoiesis and leading to the occurrence of MDS phenotypes [9]. Gene mutations involving RNA splicing, DNA methylation and DNA repair play important roles in the progression of MDS [10]. However, the effects of IOL on the immune function, gene mutations and chromosomes of MDS patients need to be further studied. Therefore, this study aimed to evaluate the clinical characteristics, bone marrow hematopoiesis, immune function, gene mutations, survival, and prognostic factors of newly diagnosed patients with IOL MDS, to provide clinical evidence for the impact of IOL on the immunity and prognosis of MDS patients.

## Patients and methods

### Patients

This study conducted a retrospective analysis of 144 MDS patients diagnosed in the Hematology Department of Tianjin Medical University General Hospital from March 2019 to December 2023. The median age of the patients was 64 (22–89) years, including 98 males and 46 females. The diagnosis of MDS patients was based on 2016 WHO diagnostic criteria and the IPSS-R (Revised international prognostic scoring system) scores [11]. Patients were categorized into the IOL group (ferritin > 1000 ng/mL) and the NIOL group (ferritin ≤ 1000 ng/mL). Blood transfusion dependence with a heavy transfusion burden (HTB) is defined as 8 RBC units in a 16-week period according the latest IWG 2018 criteria [12].

### Immunological function detection by flow cytometry

For T cell subpopulation detection: a six-color TBNK reagent kit was used to analyze peripheral blood T cell subpopulations: CD3-FITC, CD4-PE-Cy7, CD8-APC-Cy7, CD16 + 56 PE, CD19-APC, and CD45-PerCP-Cy5.5 mixed together with 5 µL of antibodies. After mixing with 50 µl of peripheral blood, the solution was incubated in the dark for 15 min. Then, 400 µL of hemolysin was added for another 10 min incubation in the dark. The samples were then mixed and analyzed by flow cytometry carried by BD FACS Canto II.

For DC cell subpopulation detection: 5 µL CD16-FITC, CD1c-PerCP-Cy5.5, lineage-PE, CD11c-PE-Cy7, Clec9A-APC, CD123-APC-Cy7, HLA-DR-BV421 and CD45-AmCyan antibodies were added to 200 µL of peripheral blood and incubated in the dark for 15 min. Then, 2 mL hemolysin was added and incubated for another 15 min. The mixture was centrifuged at 1500 r/min and the supernatant was discarded. In addition, 1 mL PBS (Phosphate Buffer Saline) was added and mixed, followed by another centrifugation step to remove the supernatant. Finally, 200 µL of PBS was added, then analyzed by flow cytometry carried by BD FACS Canto II.

Cytokine detection: Cytokine combined detection Kit (item No. P110100203) was used to detect the cytokine levels in MDS patients, which is based on the immunofluorescence technology. Through seven capture microspheres with different fluorescence intensifications in the capture microsphere mixture, the surfaces of the capture microspheres are, respectively, coated with specific antibodies of Interleukin (IL)−2, IL-4, IL-6, IL-10, TNF-α, IFN-γ, and IL-17A. The capture microspheres specifically bind to these seven cytokines in the sample to be tested, respectively, and then combine with the fluorescent detection reagent labeled with PE to form a double antibody sandwich complex formed by the capture microspheres, the sample to be tested and the detection antibody. By analyzing the fluorescence intensity of the double antibody sandwich complex, the content of cytokines in the sample to be tested can be obtained. We did the experiment referring to the instructions of the reagent kit. 25 µL peripheral blood was mixed with 25 µL fluorescent reagent C and 25 µL microspheres and incubated at room temperature in the dark for 2.5 h. After mixing with 1 mL PBS and centrifuging to remove the supernatant, the sample was resuspended in 1 mL PBS, mixed and analyzed by flow cytometry carried by BD FACS Canto II.

### Reading bone marrow smears

The bone marrow smears were stained with Wright-Giemsa. The number of megakaryocytes in the entire bone marrow smear were counted with a light microscope at × 100 magnification, and 200 nucleated cells were counted with a light microscope at × 1000 magnification. The numbers of granulocytes, immature red blood cells, lymphocytes, monocytes, plasma cells and other cells were counted, respectively. The proportion of the cells in the bone marrow smear was calculated, and the cell morphology was described. Two bone marrow smear reading experts counted the same bone marrow smear and calculated the average value.

### Gene mutation analysis

Heparin anticoagulant aseptic bone marrow 5 ml was extracted from MDS patients and centrifuged at 1500 r/min for 10 min. The bone marrow mononuclear cells (BMMNCs) from MDS patients were isolated by centrifugation in a Ficoll gradient. Intracellular DNA was extracted from the cells for sequencing. Next-generation sequencing was employed to screen for gene mutations in BMMNCs from MDS patients, using an Ion Torrent PGM sequencer/Illumina sequencer. Based on the NCBI database, different intron splicing on a gene segment can generate different transcript IDs. The gene mutations list is provided in the Supplementary Material (Table S1).

### Treatment

For the lower risk MDS (IPSS-R ≤ 3.5) patients, 25 cases with severe anemia received blood transfusions (10 cases) or combined with erythropoietin (EPO) (14 cases)/Luspatercept (1 case), 3 cases with 5q- received lenalidomide, 10 cases with symptomatic thrombocytopenia or granulocytopenia or blast cell proliferation received hypomethylating agents (4 cases azacitidine (AZA), 6 cases decitabine).

For the higher risk MDS (IPSS-R > 3.5), 24 cases with severe anemia received blood transfusions (9 cases) or combined with EPO (13 cases)/Luspatercept (2 cases), 46 cases received hypomethylating agents (22 cases AZA, 24 cases decitabine), 34 cases received AZA combined with monoclonal antibody/chemotherapy (23 cases Venetoclax, 5 cases CD47 monoclonal antibody, 4 cases programmed death-1 (PD-1) monoclonal antibody, 1 case TIM3 monoclonal antibody, 1 case CAG [clarithromycin, cytarabine, G-CSF)], and 2 cases received allogeneic hematopoietic stem cell transplantation.

### Follow-up

Patients were followed up through hospitalization records, outpatient records, and phone calls until June 2024. The median survival time was calculated from the time of diagnosis to time of death or last follow-up visit. Eight patients were lost during the follow-up (5.56%) and the median follow-up duration was 13.5 (0.1–63) months.

### Statistical analysis

Results were expressed as mean ± standard deviation or median (minimum, maximum). For normally distributed data, independent sample *t* tests were used. Non-normally distributed data were compared using non-parametric Wilcoxon tests. For categorical data, comparisons were made using Pearson's chi-squared test, continuity correction, or Fisher's exact test. Univariate and multivariate survival analyses were performed using Cox regression models. SPSS version 27 was performed for statistical analysis, and *p* values < 0.05 were considered statistically different.

## Results

### Clinical features of MDS patients

144 patients with primary MDS were included, of which 54 were in IOL group. The median age of the IOL group was 61.0 (23–85) years, with 40 males and 14 females, and the median ferritin level was 1870.09 (1055.12–5730.97) ng/ml. The NIOL group comprised 90 patients, with a median age of 64.5 (22–89) years, consisting of 58 males and 32 females. The median ferritin level was 448.81 (36.55–997.96) ng/ml. The proportion of blood transfusion dependence with HTB before diagnosis in the IOL group (57.41%) was significantly higher than that in the NIOL group (8.89%) (p < 0.05). IOL MDS patients had higher ECOG scores compared with NIOL group (p < 0.05), indicating a poorer performance status in IOL MDS patients. No significant differences were found between the groups in terms of gender, age, WHO 2016 classification, or IPSS-R score (p > 0.05) (Table 1).

**Table 1:** Characteristics of patients with MDS

	IOL MDS (*n* = 54)	NIOL MDS (*n* = 90)	T/Z/χ^2^	*p* value
Age	61.0(23–85)	64.5(22–89)	Z = − 1.751	0.08
Age ≥ 60 years	27(50.00%)	61(67.78%)	χ^2^ = 4.488	0.052
Gender (male/female)	40/14	58/32	χ^2^ = 1.440	0.23
Ferritin (ng/mL)	1870.09 (1055.12–5730.97)	448.81 (36.55–997.96)	Z = − 10.048	< 0.001
HTB	31(57.41%)	8(8.89%)	χ^2^ = 40.231	< 0.001
WHO 2016				
MDS–SLD	2(3.70%)	2(2.22%)	χ^2^ = 0.014	0.908
MDS–MLD	6(11.11%)	7(7.78%)		
MDS–RS	10(18.52%)	16(17.78%)		
MDS–5q-	1(1.85%)	3(3.33%)		
MDS–EB-1	10(18.52%)	23(25.56%)		
MDS–EB-2	23(42.59%)	37(41.11%)		
MDS-u	2(3.70%)	2(2.22%)		
IPSS-R				
≤ 1.5	0(0.00%)	2(2.22%)	χ^2^ = 1.579	0.209
> 1.5– ≤ 3	7(12.96%)	15(16.67%)		
> 3– ≤ 4.5	11(20.37%)	16(17.78%)		
> 4.5– ≤ 6	10(18.52%)	25(27.77%)		
> 6	26(48.15%)	32(35.56%)		
ECOG				
0–1	10(18.52%)	41(45.56%)	χ^2^ = 10.786	0.001
≥ 2	44(81.48%)	49(54.44%)		

### Correlation between IOL and blood cell levels and organ function in patients with MDS

By comparing blood cell levels of MDS patients, we found that the IOL MDS patients had lower white blood cell count, hemoglobin and platelet count, especially in white blood cell count and hemoglobin in IOL MDS compared with NIOL MDS (p < 0.05). In addition, we found that the proportion of EPO > 500 mU/mL in the IOL group was increased (p > 0.05). We analyzed the correlation between IOL and organ function in MDS patients. We found both alanine aminotransferase and glucose levels in IOL MDS group were significantly higher than those in the NIOL MDS group (p < 0.05). However, no clear differences were observed in lipid levels, renal function, and thyroid function between the groups (p > 0.05), indicating that IOL may damage liver and pancreatic function in patients with MDS (Table 2).

**Table 2:** Correlation between IOL and blood cell levels and organ function in patients with MDS

	IOL MDS	NIOL MDS	T/Z/χ^2^	*p* value
WBC(×10^9^/L)	2.19(0.20–25.99)	2.66(0.38–21.51)	Z = − 2.237	0.025
ANC(×10^9^/L)	0.99(0.05–78.80)	1.32(0.01–26.90)	Z = − 1.657	0.097
Hb(g/L)	61.00 (39.00–95.00)	69.50 (31.0–129.0)	Z = − 2.440	0.016
EPO > 500(mU/mL)	20(68.97%)	23(45.10%)	χ^2^ = 4.236	0.061
PLT(×10^9^/L)	42.00 (1.00–499.00)	60.00 (3.00–534.00)	Z = − 1.577	0.115
ALT(U/L)	24.00 (8.00–590.00)	15.00 (4.00–99.00)	Z = − 3.110	0.002
Cr(umol/L)	62.50 (28.00–147.00)	62.00 (27.00–566.00)	Z = − 0.305	0.760
GLU(mmol/L)	5.80(3.40–13.80)	5.20(2.80–17.40)	Z = − 2.074	0.038
TC(mmol/L)	3.50 ± 0.88	3.34 ± 0.88	t = 0.612	0.544
TG(mmol/L)	1.23 ± 0.47	1.26 ± 0.38	t = − 0.251	0.803
HDL-C(mmol/L)	0.75 ± 0.28	0.86 ± 0.36	t = − 1.078	0.287
LDL-C(mmol/L)	2.13(1.08–4.52)	1.94(0.84–7.90)	Z = − 0.587	0.557
FT3(pmol/L)	3.25 ± 0.85	3.36 ± 0.82	t = − 0.544	0.588
FT4(pmol/L)	11.80 ± 1.85	12.07 ± 1.79	t = − 0.610	0.544
TSH(uIU/mL)	1.36(0.09–6.42)	1.34(0.32–15.18)	Z = − 0.343	0.731
β_2_-MG(mg/L)	2.27(1.07–9.78)	2.18(0.81–6.79)	Z = − 0.762	0.446
LDH(U/L)	244.50 (111.10–1699.00)	211.00 (103.00–1473.00)	Z = − 1.424	0.154
CRP(mg/dL)	4.15 (0.26–150.25)	2.49 (0.10–64.25)	Z = − 1.48	0.139

### The effect of IOL on hematopoiesis in patients with MDS

The study above indicated that blood cell levels in IOL MDS patients were lower. Therefore, we analyzed the effects of IOL on bone marrow hematopoiesis. We found the proportion of blast cells in the bone marrow of IOL MDS patients was slightly higher than that in NIOL MDS group, whereas the proportion of erythroid cells was significantly lower. Concurrently, the number of megakaryocytes in the IOL MDS group was notably lower than that in NIOL MDS group (p < 0.05). However, no significant difference was found in the proportion of hematopoietic dysplasia (p > 0.05) (Table 3).

**Table 3:** Effect of IOL on bone marrow hematopoiesis in patients with MDS

	IOL MDS (*n* = 54)	NIOL MDS (*n* = 90)	T/Z/χ^2^	*p* value
Blast cells (%)	6.00 (0.00–19.50)	5.50 (0.00–19.50)	Z = − 0.257	0.797
The proportion of bone marrow myeloid cells (%)	40.31 ± 19.01	40.96 ± 18.10	t = − 0.201	0.841
The proportion of bone marrow erythroid cells (%)	21.00 (0.00–68.00)	28.00 (2.00–91.50)	Z = − 2.209	0.027
The counts of Megakaryocyte	7.00 (0.00–200.00)	41.00 (0.00–1100.00)	Z = − 3.399	< 0.001
Myeloid dysplasia	11(20.37%)	23(25.84%)	χ^2^ = 0.555	0.545
Erythroid dysplasia	11(20.37%)	22(24.72%)	χ^2^ = 0.358	0.683
Megakaryocyte dysplasia	2(3.70%)	6(6.74%)	Fisher	0.710

### The correlation between IOL and immune function in patients with MDS

Immune abnormalities play a vital role in the hematopoiesis in MDS. We analyzed the effects of IOL on T cells, DCs and cytokines in MDS patients. We found that IL-6 levels were significantly higher in IOL MDS patients compared to NIOL MDS patients (p < 0.05). The plasmacytoid DC (pDC)/DC and conventional type 2 DC (cDC2)/conventional DC (cDC) ratios in the IOL MDS group were significantly lower than those in NIOL MDS group, whereas the CD16^+^DC/cDC ratio in IOL MDS patients was significantly higher (p < 0.05). However, no statistically significant differences were found between them in the T-cell subsets (Table 4).

**Table 4:** Correlation between IOL and immune function in patients with MDS

	IOL MDS	NIOL MDS	T/Z/χ^2^	*p* value
CD3^+^ T cells (%)	76.41 (39.84–96.39)	75.73 (15.83–94.41)	Z = − 0.227	0.820
CD3^+^ CD8^+^ T cells(%)	26.54 (11.24–47.24)	25.71 (4.71–68.64)	Z = − 0.405	0.687
CD3^+^ CD4^+^ T cells (%)	43.30 ± 13.00	42.32 ± 10.78	t = 0.482	0. 631
CD16^+^ CD56^+^ NK cells(%)	12.88 (0.41–53.67)	14.10 (2.66–81.08)	Z = − 0.357	0. 720
CD19^+^ B cells(%)	7.54 (0.76–43.72)	8.23 (1.41–52.62)	Z = − 0.129	0.898
CD4^+^/CD8^+^ T cells	1.64 (0.15–4.92)	1.67 (0.33–7.71)	Z = − 0.633	0.527
IL-2(pg/mL)	1.74 (0.00–5.96)	1.54 (0.00–24.34)	Z = − 0.194	0.846
IL-4(pg/mL)	2.75 (0.01–142.35)	1.58 (0.00–125.43)	Z = − 1.164	0.244
IL-6(pg/mL)	8.66 (1.26–131.4)	5.06 (0.64–101.9)	Z = − 3.400	< 0.001
IL-10(pg/mL)	3.48 (0.32–36.55)	3.11 (0.06–113.00)	Z = − 0.562	0.574
TNF-α(pg/mL)	2.04 (0.00–8.71)	1.97 (0.00–29.72)	Z = − 0.363	0. 716
IFN-γ(pg/mL)	1.74 (0.05–10.97)	1.74 (0.01–37.87)	Z = − 0.814	0. 415
IL-17A(pg/mL)	1.34 (0.00–30.83)	2.47 (0.01–34.62)	Z = − 0.356	0.722
DC (%)	0.50 (0.10–3.20)	0.50 (0.03–11.50)	Z = − 0.147	0.886
pDC/DC (%)	1.90 (0.00–63.00)	4.60 (0.00–73.60)	Z = − 2.000	0.045
cDC/DC (%)	42.83 ± 27.14	47.98 ± 28.84	t = − 0.857	0.394
CD16^+^ DC/cDC (%)	50.80 (0.00–97.30)	28.50 (0.00–97.90)	Z = − 2.253	0.024
cDC2/cDC (%)	26.15 (0.00–95.50)	40.70 (0.00–97.40)	Z = − 2.072	0.038
cDC1/cDC (%)	1.10 (0.00–75.00)	0.70 (0.00–33.30)	Z = − 0.490	0.624

### The correlation between IOL and chromosomes in patients with MDS

Patients with MDS may exhibit various chromosomal abnormalities, among which -Y, 5q-, 20q-, 7q-, + 8, and − 7 are the most common. We found the number of patients with a normal chromosomal karyotype was significantly lower among MDS patients with IOL. Furthermore, the incidence of 7q- was higher in IOL MDS patients (p < 0.05) (Table 5).

**Table 5:** Correlation between IOL and chromosomes in patients with MDS

	IOL MDS (*n* = 54)	NIOL MDS (*n* = 90)	T/Z/χ^2^	*p* value
-Y	2 (3.70%)	0 (0.00%)	Fisher	0.139
del(5q)	7 (12.96%)	5 (5.56%)	χ^2^ = 1.552	0.213
del20(q)	4 (7.41%)	3 (3.33%)	Fisher	0.425
del7(q)	7 (12.96%)	3 (3.33%)	Fisher	0.041
+ 8	10 (18.51%)	13 (14.44%)	χ^2^ = 0.417	0.158
+ 19	0 (0.00%)	1 (1.11%)	Fisher	1.000
i(17q)	1 (1.85%)	0 (0.00%)	Fisher	0.375
− 7	2 (3.70%)	5 (5.56%)	Fisher	0.711
inv3/t(3q)/del3(q)	1 (1.85%)	1 (1.11%)	Fisher	1.000
− 22	1 (1.85%)	2 (2.22%)	Fisher	1.000
+ 11	2 (3.70%)	0 (0.00%)	Fisher	0.139
− 13	1 (1.85%)	2 (2.22%)	Fisher	1.000
+ 9	1 (1.85%)	2 (2.22%)	Fisher	1.000
Normal	22 (40.74%)	57 (63.33%)	χ^2^ = 6.956	0.008
≥ 3 abnormalities	13 (24.07%)	18 (20.00%)	χ^2^ = 0.332	0.565

### The correlation between IOL and gene mutations in patients with MDS

Patients with MDS experience various genetic mutations. The median number of gene mutations in the IOL group was 3 (0–7), while the median number of mutations in the NIOL group was 2 (0–7). The most common gene mutations among IOL MDS patients were *ASXL1*, *RUNX1*, and *TP53*, while *ASXL1, TET2*, and *DNMT3A* gene mutations in NIOL MDS patients. The mutation rate of the *ZRSR2* gene in the IOL MDS group was markedly lower than that in NIOL MDS group (p < 0.05) (Table 6).

**Table 6:** Effect of IOL on the level of genetic mutations in patients with MDS

	IOL MDS (n = 47)	NIOL MDS (n = 84)	T/Z/χ^2^	*p* value
Normal	2 (4.26%)	5 (5.95%)	Fisher	1.000
≥ 2 mutated Gene	38 (80.85%)	65 (77.38%)	χ^2^ = 0.216	0.642
*ASXL1*	17 (36.17%)	24 (28.57%)	χ^2^ = 0.809	0.368
*DNMT3A*	6 (12.77%)	17 (20.24%)	χ^2^ = 1.162	0.281
*TET2*	7 (14.89%)	19 (22.62%)	χ^2^ = 1.131	0.288
*EZH2*	2 (4.26%)	7 (8.33%)	χ^2^ = 0.276	0.600
*SF3B1*	9 (19.15%)	11 (13.10%)	χ^2^ = 0.854	0.355
*SRSF2*	2 (4.26%)	9 (10.71%)	χ^2^ = 0.903	0.342
*U2AF1*	9 (19.15%)	13 (15.48%)	χ^2^ = 0.291	0.590
*ZRSR2*	0 (0.00%)	9 (10.71%)	χ^2^ = 5.407	0.026
*RUNX1*	10 (21.28%)	9 (10.71%)	χ^2^ = 2.711	0.100
*TP53*	10 (21.28%)	14 (16.67%)	χ^2^ = 0.428	0.513
*STAG2*	5 (10.64%)	5 (5.95%)	χ^2^ = 0.392	0.531
*NRAS*	6 (12.77%)	4 (4.76%)	χ^2^ = 3.501	0.190
*CBL*	2 (4.26%)	5 (5.95%)	Fisher	1.000
*NF1*	1 (2.13%)	4 (4.76%)	Fisher	0.654
*ETV6*	1 (2.13%)	2 (2.38%)	Fisher	1.000
*GATA2*	2 (4.26%)	2 (2.38%)	Fisher	0.618
*DDX41*	3 (6.38%)	1 (1.19%)	Fisher	0.131
*IDH*	2 (4.26%)	7 (8.33%)	χ^2^ = 0.276	0.600
*SETBP1*	4 (8.51%)	4 (4.76%)	χ^2^ = 0.230	0.632
*BCOR*	8 (17.02%)	8 (9.52%)	χ^2^ = 1.580	0.209

### The effect of IOL on survival and disease progression in MDS patients

**Fig. 1:**
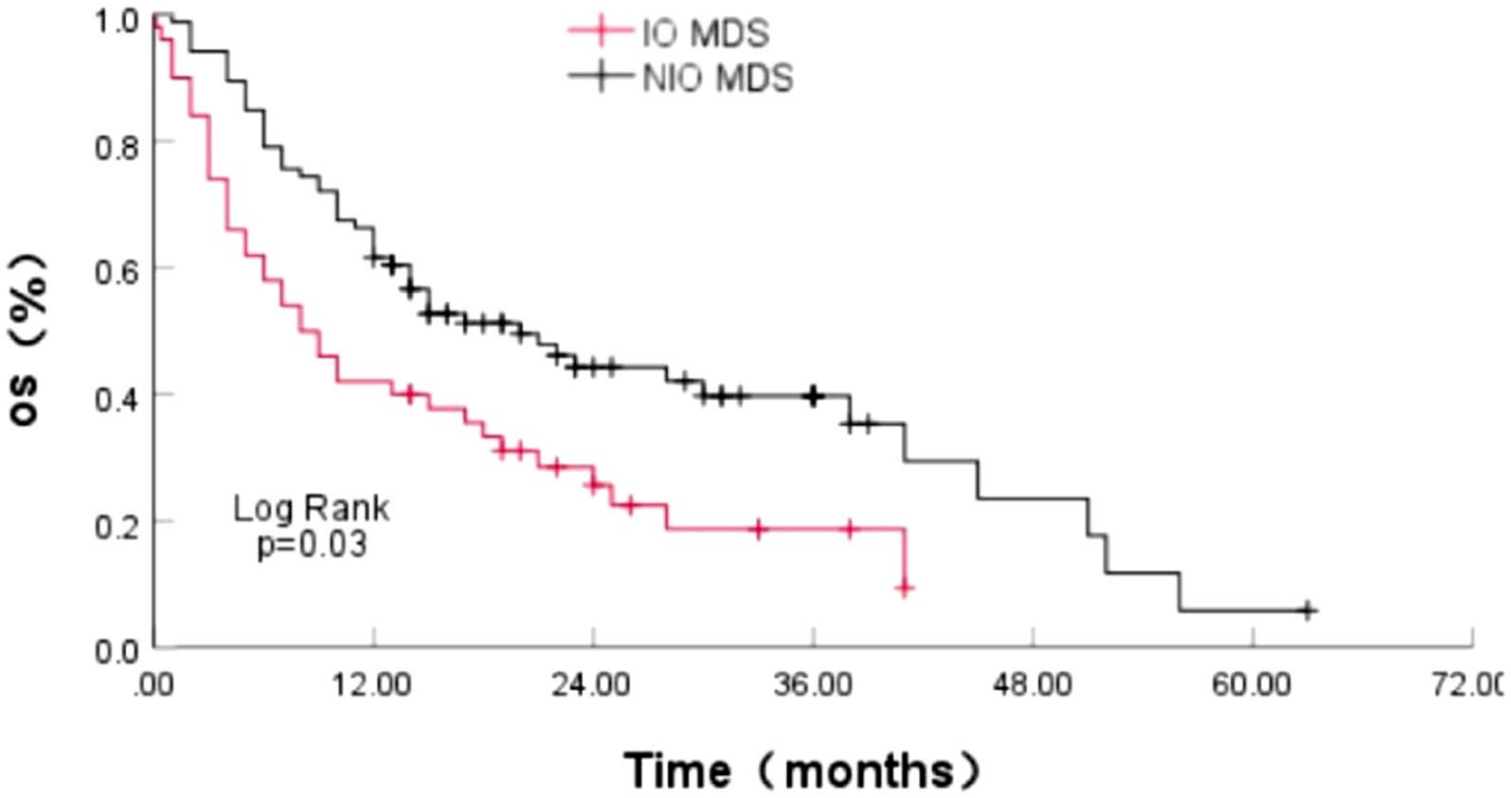
Effect of IOL on the survival of MDS patients

Our studies above indicate that IOL can affect various aspects of MDS patients. Therefore, we analyzed the effect of IOL on disease progression and survival time in patients with MDS. The median overall survival (OS) time in IOL MDS patients was 8.00 months (95% CI 4.535–11.465), which was 20.00 months in patients with NIOL MDS (95% CI 12.035–27.965). The median OS time in IOL MDS group was notably shorter than that in NIOL MDS patients, having a statistically significant variance (Log Rank p = 0.003) (Fig. 1). The proportion of disease progression or transformation to AML in IOL MDS group was 11/54 (20.37%), which was higher than that in the NIOL MDS group 17/90 (18.89%) (p < 0.05). The median progression time was 5(1–38) months, which was shorter than that in patients with NIOL MDS 8 (1–50) months (p > 0.05).

### Prognostic factors affecting patients with MDS

Cox univariate analysis was performed for factors, including gender, age, ANC, Hb, PLT, ferritin, chromosomal karyotype, and potential gene mutations affecting survival. Subsequently, factors with *p* value < 0.15 were added to the Cox multivariate analysis. The outcomes indicated that age ≥ 60y (HR = 1.502, 95% CI 1.044–2.161, p = 0.028), ferritin > 1000 ng/ml (HR = 1.452, 95% CI 1.006–2.098, p = 0.047), poor chromosomal karyotype (HR = 1.865, 95% CI 1.019–3.413, p = 0.043), *TP53* mutation (HR = 2.070, 95% CI 1.278–3.353, p = 0.003), and *RUNX1* mutation (HR = 1.707, 95% CI 1.014–2.872, p = 0.044) are independent adverse prognostic factors for MDS patients. Conversely, a good/normal chromosomal karyotype (HR = 0.627, 95% CI 0.406–0.969, p = 0.036) was an independent favorable prognostic factor for MDS patients (Table 7).

**Table 7:** Univariate and multivariate analyses of factors affecting OS in patients with MDS

Variant		Univariate Analysis		Multivariate Analysis	
		HR (95% CI)	*p* value	HR (95% CI)	*p* value
Female		1.147 (0.801–1.644)	0.453		
Age ≥ 60y		1.355 (0.953–1.928)	0.091	1.502 (1.044–2.161)	0.028
ANC < 0.8 × 10^9^/L		1.250 (0.872–1.794)	0.225		
Hb < 100 g/L		1.719 (0.977–3.021)	0.060	1.328 (0.742–2.435)	0.360
PLT < 100 × 10^9^/L		1.342 (0.904–1.993)	0.144	1.164 (0.765–1.771)	0.477
Ferritin > 1000 ng/mL		1.502 (1.054–2.139)	0.024	1.452 (1.006–2.098)	0.047
IPSS-R Cytogenetics	Very good/good	0.565 (0.394–0.811)	0.002	0.627 (0.406–0.969)	0.036
	Intermediate	1.184 (0.751–1.864)	0.465		
	Poor	0.802 (0.391–1.644)	0.547		
	Very poor	2.826 (1.689–4.729)	< 0.001	1.865 (1.019–3.413)	0.043
Mutated Genes	*TP53*	1.899 (1.185–3.042)	0.008	2.070 (1.278–3.353)	0.003
	*SRSF2*	1.050 (0.562–1.962)	0.878		
	*RUNX1*	1.499 (0.901–2.493)	0.119	1.707 (1.014–2.872)	0.044
	*NRAS*	1.014 (0.529–1.944)	0.967		
	*ASXL1*	0.927 (0.628–1.367)	0.701		
	*SF3B1*	1.175 (0.708–1.949)	0.532		
	*U2AF1*	1.025 (0.638–1.646)	0.920		
	*BCOR*	1.334 (0.806–2.207)	0.262		
	*ZRSR2*	0.862 (0.401–1.852)	0.703		

## Discussion

Iron overload occurs in various hematological disorders, and excessive iron deposition can lead to the dysfunction of multiple organs. In MDS patients, IOL is often caused by ineffective hematopoiesis and repeated blood transfusions. IOL may affect the survival of MDS patients and ICT can improve the prognosis of IOL patients [7]. Therefore, we evaluated the conditions of MDS patients from multiple perspectives, including clinical features, immune function, bone marrow hematopoiesis, gene mutations, and prognosis, to provide clinical strategies for improving the prognosis of IOL MDS patients.

MDS occurs more frequently in older patients. The median age of the MDS patients included in this study was younger than those abroad. By reviewing the data of multi-center clinical studies in China, our MDS patients were also younger compared with foreigner MDS patients. Previous studies have shown that there are genetic epigenetics racial differences in hematological tumors, including MDS [13–15]. We found that IOL MDS patients had a poorer performance according to ECOG scores, indicating a poorer performance status in IOL MDS patients. The most common causes of death in MDS patients are complications related to MDS itself and its treatment [16]. Therefore, we further analyzed the blood cell levels and organ functions of patients with MDS.

Chronic blood transfusion is the most common cause of IOL in MDS patients. However, some MDS patients have already experienced iron overload at the early stage of the disease or even before blood transfusion. Hepcidin is down-regulated in hematological diseases characterized by erythroid ineffective hematopoiesis and is considered to be associated with IOL in MDS patients [17]. We found that 57.41% of IOL MDS patients had blood transfusion dependence with a heavy transfusion burden before diagnosis. However, nearly half of the IOL MDS patients had iron overload without blood transfusion before diagnosis, which might be related to the abnormal expression of hepcidin. Iron overload can lead to hepatic and cardiac iron deposition and multiple organ dysfunction. The ALT levels in the IOL MDS patients were found to be significantly higher, which indicated that IOL can damage the liver function in MDS patients, which might lead to decreased drug tolerance in IOL MDS patients. Meanwhile, the level of blood glucose level in IOL MDS patients was significantly higher, but not on blood lipid levels and thyroid function, which was similar to the research conducted by Yassin [18]. Abnormal cellular metabolic pathways play a pivotal role in leukemia cell proliferation and survival and mediate the sustained activation of oncogenes [19]. These results suggest IOL MDS patients are more susceptible to liver function damage and elevated blood glucose levels. Severe liver failure may affect the survival of patients with MDS, whereas abnormally elevated blood glucose could promote MDS progression through tumor cell metabolism.

IOL may affect bone marrow hematopoietic function. We found that IOL MDS patients had lower levels of peripheral blood cells, especially in white blood cells and hemoglobin. Given the reduction of peripheral blood cells in IOL MDS patients, we conducted further analysis of the correlation between IOL and hematopoietic function in MDS patients. Our findings indicated the proportion of erythrocyte and megakaryocyte counts in the IOL MDS patients were significantly decreased, suggesting that the hematopoietic functions of erythrocyte and megakaryocyte were inhibited in these patients. IOL can increase the generation of reactive oxygen species (ROS), which can damage normal bone marrow stem cells in mouse models of MDS and shorten their survival [20]. Our previous research indicated that IOL increases the production of ROS in CD34^+^ and NK cells in MDS patients, and suggested that IOL may contribute to the progression of MDS [21]. These results indicate that IOL can impair bone marrow hematopoiesis in MDS patients, and the decreased hemoglobin may necessitate repeated blood transfusions, thereby exacerbating IOL. However, HTB may further inhibit erythroid hematopoiesis in the bone marrow of MDS patients. Due to the limited cases of this study, the impact of IOL on erythroid hematopoiesis in bone marrow in NTD MDS patients should be further studied in future. For diseases such as MDS, which present with bone marrow failure, IOL is undoubtedly a worsening factor.

MDS patients have various genetic and chromosomal abnormalities. Recurrent mutations involving RNA splicing, DNA methylation and DNA repair have been recognized as significant foundations for the development of MDS [10]. We found that more than 75% of MDS patients harboring two or more mutations. The *ZRSR2* gene mutation occurred exclusively in the NIOL MDS group and was significantly higher compared with the IOL MDS group. The *ZRSR2* gene is associated with RNA splicing, which involved in various signaling pathways, including myeloid differentiation and the tumor suppressor gene *PTEN* [22, 23]. Notably, in this study, mutations in *ZRSR2* gene were exclusively found in male patients. Recently, male patients with *ZRSR2* mutations were associated with an indolent clinical phenotype and improved OS through a study involving genomic analyses of 3233 patients with MDS or related disease [24]. We also found that the proportion of normal chromosome karyotypes in IOL MDS patients was markedly decreased, with the 7q chromosome abnormality being particularly prominent. The 7q anomaly is found in approximately 8% of newly diagnosed MDS patients and associated with poor OS [10]. These results suggest IOL may affect the prognosis of MDS patients by influencing genetic mutations and chromosome karyotypes.

Abnormal immune function plays a crucial role in MDS progression. Inflammation is associated with the conversion of MDS to AML and can affect the immune microenvironment in the bone marrow of leukemia patients [25]. MDS patients exhibit various gene mutations; therefore, we analyzed the immune function of MDS patients. We found that the IL-6 level in IOL MDS patients was notably higher. Previous studies have shown that the serum IL-6 level in MDS patients was significantly higher than that in the healthy control. However, there is no significant difference between the lower-risk group and the higher-risk group [26]. Recent studies have shown that the serum IL-6 level in patients with MDS–MLD was significantly higher than that in patients with MDS–SLD and MDS–RS, but there is no significant difference between different IPSS-R group. Further analysis indicates that the IL-6 level is positively correlated with the time of the first red blood cell transfusion in MDS patients [27]. Compared with healthy controls, in patients with transfusion-dependent thalassemia, the IL-6 level was significantly increased and showed a significant positive correlation with ferritin [28]. The above research results indicate that MDS patients suffered from immune abnormalities, and the high level of IL-6 is closely related to IOL MDS patients.

The predominant DCs are divided into pDC (CD11c^−^CD123^+^), conventional type I DC (cDC1) (CD11c^+^Clec9A^+^), cDC2 (CD11c^+^CD1c^+^) and monocyte-derived DC (MoDC). pDCs are the main type I interferon producing cells in humans and are able to modulate innate and adaptive immune responses [29]. In this study, we found that the pDC/DC in IOL MDS group were significantly lower. Previous studies have shown that pDC levels in lower-risk MDS patients are higher than those in normal controls, whereas higher-risk MDS patients exhibit a significant decrease in pDC levels compared to lower-risk MDS patients [30]. We further analyzed the subtype of cDC. We found that the ratio of cDC2/cDC in the IOL group was significantly lower than that in the NIOL group, whereas the CD16^+^DC/cDC ratio in the IOL MDS was significantly higher. Previous studies have shown that the ability of CD34^+^ progenitor cells differentiate to DCs in vitro in MDS patients is relatively decreased, and the defect in the number of DC precursor cells may lead to malignant clones evades immune recognition and promoting the progression of MDS to AML [31]. Previous studies have shown that CD16^+^DC is highly expressed in the peripheral blood of patients with multiple myeloma and is related to the prognosis of patients with multiple myeloma [32]. In this study, the pDC/DC ratio and cDC2/cDC ratio was significantly decreased in IOL MDS patients, indicating that IOL may participate in disease progression by affecting subpopulations of DCs in MDS patients.

IOL could affect the survival of MDS patients. We found that the OS of IOL MDS patients was significantly shorter with significantly higher progression rate. Although the progression time of MDS in IOL group was slightly shorter, and a further extended follow-up is required for confirmation. Kikuchi demonstrated that SF (serum ferritin) levels were significantly higher in patients with higher-risk MDS, and the patients in the lower SF group showed a marked increase in OS and leukemia-free survival [33]. In MDS patients, a transferrin saturation > 80% and ferritin levels > 800 µg/L were associated with a poor 5-year OS, progression-free survival, and leukemia-free survival [34]. These results confirm the effect of IOL on the survival and disease progression of MDS.

We conducted univariate and multivariate analyses of the prognosis in MDS patients, revealing that age ≥ 60 years, ferritin > 1000 ng/ml, extremely poor chromosomal karyotype, gene mutations of *TP53* and *RUNX1* were independent adverse prognostic factors for MDS patients. IOL indicates a relatively poor prognosis. ICT is associated with prolonged survival in transfusion-dependent MDS patients, and IOL is also a poor prognostic factor in MDS patients who undergo allogeneic hematopoietic stem cell transplantation [35, 36]. *TP53* and *RUNX1* gene mutations in MDS patients were associated with unfavorable clinical features and prognosis [37, 38], which were consistent with this study. Moreover, a good chromosomal karyotype is an independent favorable prognostic factor in MDS patients.

## Conclusion

Based on the findings above, MDS patients with IOL exhibit poorer performance status and partial impairment of the liver and pancreatic functions. The peripheral blood cells of IOL MDS patients were decreased, especially in white blood cells and hemoglobin, while the erythrocyte and megakaryocyte in bone marrow were significantly suppressed, indicating a more severe degree of bone marrow failure. Iron overload can lead to genomic and chromosomal instability, with a significant reduction in the *ZRSR2* gene mutation and increased incidence of 7q- chromosomal abnormalities in IOL MDS patients. Aberrations in the levels of inflammatory factors and DC subpopulations in IOL MDS patients may promote disease progression. All of mentioned above contribute to the multifaceted effects of IOL on the prognosis of MDS. Our analysis provides a direction for further in-depth research on the mechanisms related to IOL and the development of corresponding targeted therapies.

## Supplementary Information

Additional file 1. Table S1. List of gene mutations

## Data Availability

No datasets were generated or analysed during the current study.

## Abbreviations

AML: Acute myeloid leukemia
AZA: Azacitidine
BMMNCs: Bone marrow mononuclear cells
cDC1: Conventional type I dendritic cell
cDC2: Conventional type 2 dendritic cell
DC: Dendritic cell
EPO: Erythropoietin
HTB: Heavy transfusion burden
ICT: Iron chelation therapy
IL: Interleukin
IOL: Iron overload
IPSS-R: Revised international prognostic scoring system
MDS: Myelodysplastic syndrome
MoDC: Monocyte-derived dendritic cell
NIOL: Non-iron overload
OS: Overall survival
pDC: Plasmacytoid dendritic cell
PD-1: Programmed death-1
PBS: Phosphate Buffer Saline
ROS: Reactive oxygen species
SF: Serum ferritin

## References

[1] Zeidan AM, Griffiths EA, Zeidan AM, et al. To chelate or not to chelate in MDS: That is the question! Blood Rev. 2018;32(5):368–77. 29602612 10.1016/j.blre.2018.03.002

[2] Malcovati L, Porta MG, Pascutto C, Invernizzi R, Boni M, Travaglino E, et al. Prognostic factors and life expectancy in myelodysplastic syndromes classified according to WHO criteria: a basis for clinical decision making. J Clin Oncol. 2005;23(30):7594–603. 16186598 10.1200/JCO.2005.01.7038

[3] Takatoku M, Uchiyama T, Okamoto S, Kanakura Y, Sawada K, Tomonaga M, et al. Retrospective nationwide survey of Japanese patients with transfusion-dependent MDS and aplastic anemia highlights the negative impact of iron overload on morbidity/mortality. Eur J Haematol. 2007;78(6):487–94. 17391310 10.1111/j.1600-0609.2007.00842.x

[4] Mitchell M, Gore SD, Zeidan AM. Iron chelation therapy in myelodysplastic syndromes: where do we stand? Expert Rev Hematol. 2013;6(4):397–410. 23991926 10.1586/17474086.2013.814456 PMC4124619

[5] Duarte TL, Lopes M, Oliveira M, Santos AG, Vasco C, Reis JP, Antunes AR, Gonçalves A, Chacim S, Oliveira C, Porto B, Teles MJ, Moreira AC, Silva AMN, Schwessinger R, Drakesmith H, Henrique R, Porto G, Duarte D, Duarte TL, et al. Iron overload induces dysplastic erythropoiesis and features of myelodysplasia in Nrf2-deficient mice. Leukemia. 2024;38(1):96–108. 37857886 10.1038/s41375-023-02067-9

[6] An W, Feola M, Levy M, Aluri S, Ruiz-Martinez M, Sridharan A, Fibach E, Zhu X, Verma A, Ginzburg Y, An W, et al. Iron chelation improves ineffective erythropoiesis and iron overload in myelodysplastic syndrome mice. Elife. 2023;28(12): e83103. 10.7554/eLife.83103 PMC10754500 38153418

[7] Yang S, Zhang MC, Leong R, Mbuagbaw L, Crowther M, Li A, Yang S, et al. Iron chelation therapy in patients with low- to intermediate-risk myelodysplastic syndrome: a systematic review and meta-analysis. Br J Haematol. 2022;197(1):e9–11. 34927248 10.1111/bjh.17998

[8] Rodriguez-Sevilla JJ, Colla S. T-cell dysfunctions in myelodysplastic syndromes. Blood. 2024;143(14):1329–43. 38237139 10.1182/blood.2023023166

[9] Lynch OF, Calvi LM. Immune dysfunction, cytokine disruption, and stromal changes in myelodysplastic syndrome: a review. Cells. 2022;11(3):580. 35159389 10.3390/cells11030580 PMC8834462

[10] Shallis RM, Ahmad R, Zeidan AM, Shallis RM, et al. The genetic and molecular pathogenesis of myelodysplastic syndromes. Eur J Haematol. 2018;101(3):260–71. 29742289 10.1111/ejh.13092

[11] Greenberg PL, Tuechler H, Schanz J, et al. Revised international prognostic scoring system for myelodysplastic syndromes. Blood. 2012;120:2454–65. 22740453 10.1182/blood-2012-03-420489 PMC4425443

[12] Platzbecker U, Fenaux P, Adès L, et al. Proposals for revised IWG 2018 hematological response criteria in patients with MDS included in clinical trials. Blood. 2019;133(10):1020–30. 30404811 10.1182/blood-2018-06-857102 PMC7042664

[13] Huang H, Xu C, Gao J, et al. Severe ineffective erythropoiesis discriminates prognosis in myelodysplastic syndromes: analysis based on 776 patients from a single centre. Blood Cancer J. 2020;10(8):83. 32801296 10.1038/s41408-020-00349-4 PMC7429953

[14] Yan X, Wang L, Jiang L, et al. Clinical significance of cytogenetic and molecular genetic abnormalities in 634 Chinese patients with myelodysplastic syndromes. Cancer Med. 2021;10(5):1759–71. 33609081 10.1002/cam4.3786 PMC7940222

[15] Huang H, Wu J, Qin T, Xu Z, et al. Is race important in genomic classification of hematological neoplasms? Hematol Oncol. 2021;39(5):728–32. 34392561 10.1002/hon.2909

[16] Stempel JM, Podoltsev NA, Dosani T, Stempel JM, et al. Supportive care for patients with myelodysplastic syndromes. Cancer J. 2023;29(3):168–78. 37195773 10.1097/PPO.0000000000000661

[17] Parisi S, Finelli C. Prognostic factors and clinical considerations for iron chelation therapy in myelodysplastic syndrome patients. J Blood Med. 2021;3(12):1019–30. 10.2147/JBM.S287876 PMC8651046 34887690

[18] Yassin MA, Soliman A, De Sanctis V, Hmissi SM, Abdulla MAJ, Ekeibed Y, Ismail O, Nashwan A, Soliman D, Almusharaf M, Hussein R, Yassin MA, et al. The impact of iron overload in patients with acute leukemia and myelodysplastic syndrome on hepatic and endocrine functions. Acta Biomed. 2018;89(3):18–22. 29633728 10.23750/abm.v89i3-S.7213 PMC6179097

[19] Rashkovan M, Ferrando A. Metabolic dependencies and vulnerabilities in leukemia. Genes Dev. 2019;33:1460–74. 31676734 10.1101/gad.326470.119 PMC6824464

[20] Jin X, He X, Cao X, Xu P, Xing Y, Sui S, Wang L, Meng J, Lu W, Cui R, Ni H, Zhao M. Iron overload impairs normal hematopoietic stem and progenitor cells through reactive oxygen species and shortens survival in myelodysplastic syndrome mice. Haematologica. 2018;103(10):1627–34. 29903757 10.3324/haematol.2018.193128 PMC6165791

[21] Hua Y, Wang C, Jiang H, Wang Y, Liu C, Li L, Liu H, Shao Z, Fu R. Iron overload may promote alteration of NK cells and hematopoietic stem/progenitor cells by JNK and P38 pathway in myelodysplastic syndromes. Int J Hematol. 2017;106(2):248–57. 28405919 10.1007/s12185-017-2237-x

[22] Gangat N, Patnaik MM, Tefferi A, Gangat N, et al. Myelodysplastic syndromes: contemporary reviewand how we treat. Am J Hematol. 2016;91(1):76–89. 26769228 10.1002/ajh.24253

[23] Tseng CC, Obeng EA, Tseng CC, et al. RNA splicing as a therapeutic target in myelodysplastic syndromes. Semin Hematol. 2024;24:00121–5. 10.1053/j.seminhematol.2024.10.005 39542752

[24] Bernard E, Hasserjian RP, Greenberg PL, et al. Molecular taxonomy of myelodysplastic syndromes and its clinical implications. Blood. 2024;144(15):1617–32. 38958467 10.1182/blood.2023023727 PMC11487646

[25] Balandrán JC, Lasry A, Aifantis I, Balandrán JC, et al. The role of inflammation in the initiation and progression of myeloid neoplasms. Blood Cancer Discov. 2023;4(4):254–66. 37052531 10.1158/2643-3230.BCD-22-0176 PMC10320626

[26] Kittang AO, Sand K, Brenner AK, Rye KP, Kittang BØ, et al. The systemic profile of soluble immune mediators in patients with myelodysplastic syndromes. Int J Mol Sci. 2016;17(7):1080. 27399678 10.3390/ijms17071080 PMC4964456

[27] Topping J, Taylor A, Nadat F, et al. Inflammatory profile of lower risk myelodysplastic syndromes. Br J Haematol. 2024;205(3):1044–54. 38772913 10.1111/bjh.19530

[28] Vinchi F, Sparla R, Passos ST, et al. Vasculo-toxic and pro-inflammatory action of unbound haemoglobin, haem and iron in transfusion-dependent patients with haemolytic anaemias. Br J Haematol. 2021;193(3):637–58. 33723861 10.1111/bjh.17361 PMC8252605

[29] Roussel X, GarnacheOttou F, Renosi F. Plasmacytoid Dendritic Cells, a Novel Target in Myeloid Neoplasms. Cancers (Basel). 2022;14(14):3545. 35884612 10.3390/cancers14143545 PMC9317563

[30] Van Leeuwen-Kerkhoff N, Westers TM, Poddighe PJ, et al. Reduced frequencies and functional impairment of dendritic cell subsets and non-classical monocytes in myelodysplastic syndromes. Haematologica. 2022;107(3):655–67. 33567812 10.3324/haematol.2020.268136 PMC8883570

[31] Micheva I, Thanopoulou E, Michalopoulou S, et al. Impaired generation of bone marrow CD34-derived dendritic cells with low peripheral blood subsets in patients with myelodysplastic syndrome. Br J Haematol. 2004;126(6):806–14. 15352984 10.1111/j.1365-2141.2004.05132.x

[32] Xu P, Li Y, Zhuang X, et al. Changes in immune subsets during chemotherapy as prognosis biomarkers for multiple myeloma patients by longitudinal monitoring. Immunol Res. 2024;72(5):1185–97. 39254909 10.1007/s12026-024-09521-5

[33] Kikuchi S, Kobune M, Iyama S, Sato T, Murase K, Kawano Y, Takada K, Ono K, Hayashi T, Miyanishi K, Sato Y, Takimoto R, Kato J. Prognostic significance of serum ferritin level at diagnosis in myelodysplastic syndrome. Int J Hematol. 2012;95(5):527–34. 22407873 10.1007/s12185-012-1048-3

[34] Teichman J, Geddes M, Zhu N, et al. High transferrin saturation predicts inferior clinical outcomes in patients with myelodysplastic syndromes. Haematologica. 2023;108(2):532–42. 35979720 10.3324/haematol.2022.280723 PMC9890030

[35] Shenoy N, Vallumsetla N, Rachmilewitz E, et al. Impact of iron overload and potential benefit from iron chelation in low-risk myelodysplastic syndrome. Blood. 2014;124(6):873–81. 24923296 10.1182/blood-2014-03-563221 PMC4467862

[36] Zhao W, Zeng X, Pan D, et al. The impact of granulocyte colony-stimulating factor and decitabine-containing conditioning in myelodysplastic syndrome patients with iron overload undergoing allogeneic hematopoietic stem cell transplantation: a retrospective study. Ther Adv Hematol. 2024;21(15):20406207241292452. 10.1177/20406207241292451 PMC11580088 39574481

[37] Montoro MJ, Palomo L, Haferlach C, et al. Influence of TP53 gene mutations and their allelic status in myelodysplastic syndromes with isolated 5q deletion. Blood. 2024;144(16):1722–31. 39074355 10.1182/blood.2024023840

[38] Falantes JF, Márquez-Malaver FJ, Carrillo E, et al. SF3B1, RUNX1 and TP53 Mutations significantly impact the outcome of patients with lower-risk myelodysplastic syndrome. Clin Lymphoma Myeloma Leuk. 2022;22(12):e1059–66. 36117041 10.1016/j.clml.2022.08.012

