# Supplementary material for: Clinical features and outcomes of myelodysplastic syndrome patients with iron overload: a single-center retrospective study
Source: Eur J Med Res. 2025 Jul 9;30:600. doi: 10.1186/s40001-025-02848-1 (PMC12239465; doi:10.1186/s40001-025-02848-1)
Supplement: Supplementary file 1 — Additional file 1. Table S1. List of gene mutations [file 40001_2025_2848_MOESM1_ESM.docx]

**Table S1. List of gene mutations**

| *AKNRD26* | *ASXL1* | *ATG2B* | *BCOR* | *BCORL1* | *BLM* |
| --- | --- | --- | --- | --- | --- |
| *BRCA1* | *BRCA2* | *CALR* | *CBL* | *CEBPA* | *CSF3R* |
| *DDX41* | *DKC1* | *DNMT3A* | *ELANE* | *ETV6* | *EZH2* |
| *FLT3* | *GATA2* | *GFI1* | *GSKIP* | *HAX1* | *IDH1* |
| *IDH2* | *JAK2* | *KIT* | *KMT2A* | *KRAS* | *MPL* |
| *NF1* | *NPM1* | *NRAS* | *PDGFRA* | *PHF6* | *PIGA* |
| *PPM1D* | *PTPN11* | *RUNX1* | *SBDS* | *SETBP1* | *SF3B1* |
| *SH2B3* | *SRP72* | *SRSF2* | *STAG2* | *STAT3* | *TERC* |
| *TERT* | *TET2* | *TP53* | *U2AF1* | *WT1* | *ZRSR2* |
